# Attitudes towards dementia prevention in Austria—Results of an exploratory study

**DOI:** 10.1007/s00391-025-02433-5

**Published:** 2025-04-02

**Authors:** Paul Pürcher, Margit Höfler, Stefanie Auer

**Affiliations:** 1 Department for Dementia Research and Care Science, University of Continuing Education Krems, Dr.-Karl-Dorrek-Straße 30, 3500 Krems, Austria; 2 Department of Medical Psychology, Center for Public Health, Medical University of Vienna, Kinderspitalgasse 15, 1090 Vienna, Austria; 3 Unit Public Mental Health, Department of Social and Preventive Medicine, Center for Public Health, Medical University of Vienna, Kinderspitalgasse 15, 1090 Vienna, Austria; 4 Department of Psychology, University of Graz, Universitätsplatz 2/DG, 8010 Graz, Austria

**Keywords:** Brain health, Cultural adaption, Awareness building, Individualized prevention strategies, Workshop, Hirngesundheit, Kulturelle Anpassung, Bewusstseinsbildung, Individualisierte Präventionsstrategien, Workshop

## Abstract

**Background:**

Changes in lifestyle could reduce dementia cases by 45%. Successful international prevention initiatives show the importance of involving primary target groups early in the process of program development.

**Objective:**

The aim of this study was to explore (1) the knowledge about dementia/brain health, (2) offers of activities related to dementia prevention in the community and (3) the feasibility of introducing the experience of art as a brain health-promoting method in a sample of community-dwelling older persons.

**Material and methods:**

Senior volunteers from the community were invited to a workshop and a follow-up telephone interview. During the workshop, participants received expert input on dementia/brain health followed by several group discussion rounds using a “World Café” approach. After 6 weeks, participants were invited to a structured telephone interview.

**Results:**

A total of 26 persons participated in the workshop, 20 of whom (mean age: 70.60 years) took part in the telephone interview. The workshop data revealed 4 main needs: (1) more information on dementia/brain health (2) a broader offer of activities for physical/mental stimulation in the community (3) the definition of a “brain health strategy”, and (4) development of specific services to experience art. The telephone interviews revealed a high motivation to start with dementia prevention but appropriate services are missing in the communities.

**Conclusion:**

Our findings provide first insights into attitudes towards dementia prevention/brain health in an Austrian sample of senior citizens. People need information about the potential of dementia prevention and specific services need to be developed in the communities.

## Abstract (de)

**Hintergrund:**

Eine Optimierung des Lebensstils könnte 45 % der Demenzfälle verhindern. Erfolgreiche internationale Präventionsinitiativen zeigen die Wichtigkeit einer frühen Einbindung der primären Zielgruppe bei der Entwicklung von Programmen.

**Ziel der Arbeit:**

Ziel dieser Untersuchung war die Exploration von 3 Bereichen: (1) Wissen älterer Personen über Demenz/Hirngesundheit, (2) Angebote in den Gemeinden, (3) mögliches Einbeziehen von Kunst als Methode zur Erhaltung der Hirngesundheit.

**Material und Methoden:**

Ältere Personen aus der Bevölkerung wurden zu einem Workshop und Telefoninterview eingeladen. Im Workshop erhielten sie Inputs von Expert:innen zu Demenz/Hirngesundheit und diskutierten in einem *„World-Café“-Format*. Nach 6 Wochen wurden die Teilnehmer:innen zu einem strukturierten Telefoninterview eingeladen.

**Ergebnisse:**

Insgesamt nahmen 26 ältere Personen am Workshop teil, von diesen nahmen 20 Personen (mittleres Alter: 70,60 Jahre) am Telefoninterview teil. Insgesamt wurden 4 Bedarfsbereiche identifiziert: (1) mehr Information zu Demenz/Hirngesundheit, (2) breiteres Angebot an Aktivitäten in den Gemeinden, (3) die Definition einer „Hirngesundheitsstrategie“ und (4) Erarbeitung spezifischer Angebote, die Kunst für alle erlebbar macht. Die Ergebnisse des Telefoninterviews zeigten eine hohe Motivation, mit Demenzprävention zu beginnen, aber Unzufriedenheit mit aktuellen Angeboten.

**Diskussion:**

Unsere Ergebnisse geben erste Einblicke zu Ansichten älterer Personen in Österreich hinsichtlich Demenzprävention/Hirngesundheit. Information zum Potenzial von Demenzprävention ist wichtig, ebenso konkrete Angebote in den Gemeinden.

## Introduction

Recent research identified 14 risk factors for dementia that apply to different phases of life [1] and to different world regions [2]. A reduction of these risk factors could prevent 45% of dementia cases [1]. These recent scientific developments need to be translated into interventions that reach as many population segments as possible. Therefore, it is important to explore the needs of the target population before planning prevention programs.

## Background

More than 55 million people worldwide are living with dementia and this number will grow rapidly over the next years [3]. Dementia is a cost-intensive disease [4, 5] with a large impact on the well-being of the affected person [6] as well as on the well-being of their formal and informal caregivers [7]. Approximately 80% of the public are concerned about developing dementia at some point, and one in four persons thinks that there is nothing that can be done to prevent this disease [8]. Hence, it is necessary to enhance knowledge on dementia and dementia prevention in the population.

Different dementia prevention initiatives have already been developed around the world starting in the early 2000s [9–11]. While these studies focused on single domain interventions and revealed controversial results, the worldwide first statistically significant randomized controlled trial concerning dementia prevention (Finnish Geriatric Intervention Study to Prevent Cognitive Impairment and Disability, FINGER study) demonstrated that a multidomain treatment consisting of physical activity, cognitive stimulation, nutritional advice and the monitoring of medical risk factors could successfully prevent cognitive decline [12]. Based on these findings, the World Health Organization (WHO) urged all countries to develop dementia prevention strategies [13]: however, it has been stressed that such prevention initiatives must be culturally adapted to different settings [14]. Also, museums have been recently suggested to contribute to cognitive health among older adults [15, 16] and might therefore be valuable resources when planning prevention programs.

Knowledge about the potential of dementia prevention in the general population has been described to be low [17]. Interviews with older adults not only showed that their knowledge of the cause and risk factors of cognitive disorders and prevention was limited and superficial but at the same time people also voiced a need for up to date, reliable and practical information and advice [17]. The aim of the current study was therefore to explore in greater detail the knowledge about dementia and brain health but also the awareness about offers of activities related to dementia prevention in the communities in a sample of Austrian senior citizens. Moreover, as arts experience has been shown to be a potential resource for brain health [15, 16], it is also an open question how people feel about including museums and art experience into prevention programs. Hence, we were also interested in the feasibility of art/museums to encourage health-promoting behavior.

## Study design and investigation methods

### Recruitment

We contacted six senior citizen organizations and sent an invitation to participate in a workshop on dementia prevention. The invitation called for persons (all genders) from the age of 55 years onwards who were interested in brain health and were worried about their memory. Interested people registered via telephone or e‑mail. Workshop participants were also asked to participate in a follow-up telephone interview. The study was accepted by the Ethics Committee of the University for Continuing Education in Krems, Austria. The research was carried out in accordance with the Helsinki Declaration [18].

### Workshop agenda

The 1‑day workshop took place in October 2021 in the German language. In the morning session, short impulse presentations on dementia prevention were provided by five experts. The German dementia activist Helga Rohra presented a “lived experience” statement. The presentations were followed by three “World Café” [19] discussions in smaller subgroups, led by two moderators where three topics were discussed: (1) participants’ knowledge about dementia and brain health, (2) offers of activities related to dementia prevention and recommendations for potential additional activities by the participants and (3) feasibility of introducing art experience in prevention routine [16, 20]. During the guided discussions, the “World Café” moderators created posters together with the workshop participants summarizing the main discussion points. All posters were presented and discussed at the end of the discussion rounds with all workshop participants. After the workshop, a qualitative content analysis of all discussion topics was performed. The summaries of the three discussion rounds were transcribed from the posters and the discussion points were organized into categories. From these categories we identified overarching themes.

### Telephone interview

Participants were contacted via telephone 6 weeks after the workshop and asked to participate in the telephone interview. For the telephone interview, a questionnaire with five closed, one open and five demographic questions was constructed. In the closed questions, participants were first asked to rate, on a 5-point Likert scale ranging from (1) “very unsatisfied”/“very low” to (5) “very satisfied”/“very high”, their overall satisfaction with the workshop discussion and their knowledge gain. Question 2 addressed the satisfaction of the participant with dementia prevention activities in their home towns/villages. Questions 3 and 4 asked about the personal interest in art and the potential of experiencing art as stimulating method for dementia prevention. Question 5 asked about the current motivation to engage in prevention activities. Finally, question 6 (open question) explored the personal gain from the workshop in greater detail.

The demographic information consisted of age, place of residence (small town, city), highest achieved education (e.g., professional qualifications, university degree) living arrangements (alone or with partner), working status (still working, retired). All telephone interviews (duration about 20 min) were carried out by one of the authors (PP). Mean scores and standard deviations were calculated for the closed questions. The answers from the open question were summarized by the interviewer during the telephone interview and the content of these notes was categorized and overarching themes were identified.

## Results

### Workshop discussion

A total of 26 persons (4 males, 22 females) participated in the workshop and gave their verbal consent to be contacted after the workshop to participate in a follow-up interview. Most of the participants were present on-site (24 participants), while 2 persons participated online. The qualitative content analysis revealed four overarching categories: (A) information, (B) activities, (C) implementation strategies and (D) arts experience. With respect to “information”, participants emphasized a lack of information and the necessity to gain better knowledge on the topic of dementia prevention and the role of risk factors. Furthermore, participants stressed the importance of personal responsibility and availability of information within the public domain. With respect to dementia, participants noted that information for people with worries but also for the public is missing. With respect to “activities”, the development of appropriate motivational strategies (e.g., how to start prevention activities and how to maintain them) were discussed as well as the gender differences on motivational strategies (men seem to have different needs than women). The workshop participants also indicated that the activities should be personalized and based on their individual risk factor profile. The importance of intergenerational exchange was stressed to strengthen the bond between generations and to learn from each other (e.g., by organizing intergenerational projects in sports clubs and museums). When organizing activities, public meeting points with good accessibility (also by public transport) were suggested. With respect to the “implementation strategies”, participants suggested a strategy including activities for persons with dementia, caregivers, and the public (i.e., a brain health strategy). For these strategies, paternalism needs to be avoided and the difference between urban and rural environments should be considered. Above all, one of the main discussion points was that individual needs must be respected. With respect to the category accepting “arts experience” as a stimulating experience, participants voiced the preference of having age-appropriate programs in museums. During these special age-appropriate programs, the venues should be quiet (avoiding high peak visitor times in the museum). In the age-appropriate programs additional information on selected artefacts should be provided and a social program (e.g., coffee afterwards) should be organized to enable additional discussions of the arts experience. They also emphasized that participation needs to be affordable for all.

### Telephone interview

**Fig. 1:**
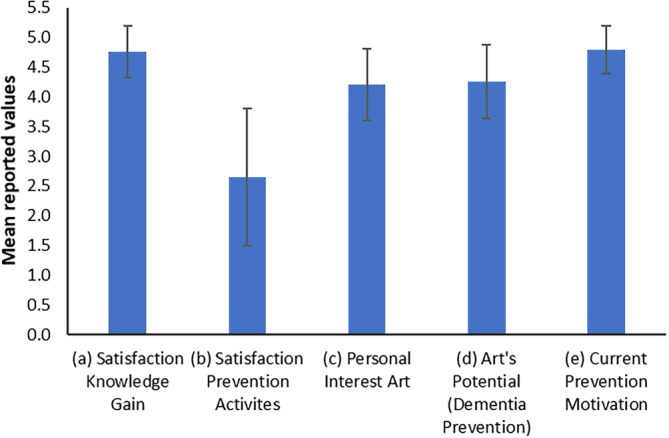
Average values regarding the attitudes towards the five closed questions asked during the telephone interview (Likert Scale 1–5; 1: Very unsatisfied/Very low, 5: Very satisfied/Very high). Error bars represent the standard deviation

Of the persons who attended the workshop 20 (4 males, 16 females) also took part in the follow-up telephone interview. The mean age of participants was 70.60 years (range: 56–85 years), 18 participants were retired and 2 participants were still formally working, 10 participants lived in communities with less than 5000 inhabitants, 3 participants in communities with more than 5000 inhabitants and 7 participants lived in Vienna with a population of around 2 million people. Of the participants 8 reported to live alone and 12 lived together with their families. Overall, participants were very satisfied with the knowledge gained from the workshop (mean Likert scale, M: 4.75, standard deviation, SD: 0.43; see Fig. 1). They reported that the offers and activities regarding dementia prevention provided in their home communities were not sufficient (M: 2.65, SD: 1.15). Both general interest for art (M: 4.2, SD: 0.60) as well as the rating of the potential of art as a method of dementia prevention (M: 4.25, SD: 0.62) were high. On average participants’ personal motivation to start with dementia prevention activities was high (M: 4.79, SD: 0.40). When asked about the personal gain of the workshop (open question), participants appreciated that dementia is being actively researched in academia and that efforts are being made to create prevention programs. They also stated that they gained knowledge that something can be actively done to prevent dementia (see Fig. 1 for an overview of the results).

## Discussion

In the current study we aimed to explore (1) the knowledge about dementia and brain health, (2) offers of activities related to dementia prevention in the community and (3) the feasibility of art/museums to encourage health-promoting behavior.

One of the main results of the workshop and the interviews was the high interest of the participants with respect to information concerning the topic of dementia and dementia prevention. This is also in line with the findings of Rosenberg et al. [17] who identified superficial knowledge on dementia and dementia prevention among their sample from Finland. As in the Austrian sample, persons requested information and practical advice. Superficial knowledge of cognitive disorders in general among vulnerable groups (for example older persons) can create fear, hopelessness and misery, which might indicate stigma [17]. Knowledge is very important when it comes to dementia prevention, and missing knowledge has also been identified as a barrier for lifestyle change [21]. During our workshop discussion it was impressive to see how relieved persons reacted to the information that something can be done against dementia. One workshop participant said: “I had no idea that I can do something to keep my brain healthy by being more active”. Another important point of discussion was the question of how to move from knowledge to action. Here we were able to identify a possible gap between motivation and actual ability to change the behavior: people were interested in the topic, wanted to adhere to preventive measures, thus showed high motivation but did not know how to get started and how to maintain the motivation. For planning such prevention activities it is therefore important to differentiate between health literacy (knowledge about health topics) and health empowerment (people in an active role when it comes to controlling their health) [22]. Despite being equally important, both areas require different methodologies and interventional approaches.

During the workshop, people also expressed a strong wish for intergenerational activities (discussion rounds, sports activities, museum visits). The participants voiced a concern that the connection between different generations might get lost if no common activities were initiated. Different generations could have the chance to learn from each other [23]. Another important observation in our sample was that men might be less receptive for activities of dementia prevention than women. In our workshop four men participated. The difference between men and women with respect to health behavior has been pointed out in the literature. For example, Hiller et al. [24] reported the results of a qualitative synthesis of 23 studies in 13 different countries concerning primary prevention and found a tendency for women to be more conscious about health and more attracted to preventive behavior compared to men; however, health behavior is a complex sociocultural challenge and further research is needed to account for all its facets [25].

During the telephone interview, participants expressed to be rather dissatisfied with the activities and offers for dementia prevention in their home communities. Many more offers should be developed to meet the individual interest. The motivation to engage in dementia prevention activities was rated very high during the telephone interview but some participants enforced the need for assistance to get started. Most of the participants were interested in art education and endorsed the idea of a high potential for art education in dementia prevention [16, 26]. Museums can be found all over the country and are an important source of knowledge and inspiration.

### Limitations

It needs to be acknowledged that the workshop participants consisted of a self-selected group of individuals who were highly motivated and interested in brain health and prevention of dementia as we solely recruited through senior organizations. We also had to limit the number of participants due to restrictions during the COVID-19 pandemic. To gain an even broader understanding, more such workshops in communities should be organized. The creation of different settings to attract people from different socioeconomic backgrounds is necessary. It also cannot be ruled out that the participants may have given socially desirable answers during the telephone interview as the questionnaire used for the telephone interview was not tested beforehand. Therefore, our results cannot be generalized and further research is necessary to gain a deeper insight into the motives and hurdles to embark on a more active lifestyle.

## Conclusion and practical recommendations

The results from the workshop and telephone interviews provide important first insights into the attitudes towards dementia prevention and brain health in an Austrian sample of senior citizens. Participants reported a big interest in the topic of dementia and dementia prevention: This information can be used to develop teaching and information material targeting the general population. Secondly, participants voiced the importance of community-based personalized prevention programs and thirdly, participants were interested in arts and saw a high potential for arts education for dementia prevention, if respective programs are age-appropriate and affordable. Further research is needed to enable a broader insight into the mechanisms of dementia prevention and how they are accepted in the population.

## Data Availability

The data of this study are available from the corresponding author upon request.

## Declarations

All studies mentioned were in accordance with the ethical standards indicated in each case.
